# Supplementary material for: EnzML: multi-label prediction of enzyme classes using InterPro signatures
Source: BMC Bioinformatics. 2012 Apr 25;13:61. doi: 10.1186/1471-2105-13-61 (PMC3483700; doi:10.1186/1471-2105-13-61)
Supplement: Addtional file 5 — The Java code to format the data files, evaluate and predict. The file enzml_java_code.tar.gz contains the Java code used to format database data to ARFF and XML formats, to execute cross and train-test (jackknife) evaluations and to record evaluation results to database. More information is included in the readme.txt file and the Javadoc files. The code can be used with a MySQL database. To use a different database software, other JDBC drivers might be required. [file 1471-2105-13-61-S5.gz › java_code/enzml2011/doc/uk/ac/ed/inf/enzml/mulan/MulanDataSetGenerator.html]

MulanDataSetGenerator


---


|  |  |  |
| --- | --- | --- |
| **Overview** \| **Package** \| **Class** \| **Use** \| **Tree** \| **Deprecated** \| **Index** \| **Help** | |  |
| **PREV CLASS**   **NEXT CLASS** | **FRAMES**    **NO FRAMES**     **All Classes** |  |
| SUMMARY: NESTED \| FIELD \| CONSTR \| METHOD | DETAIL: FIELD \| CONSTR \| METHOD |  |


---


## uk.ac.ed.inf.enzml.mulan Class MulanDataSetGenerator

```
java.lang.Object
  uk.ac.ed.inf.enzml.weka.DataSetGenerator
      uk.ac.ed.inf.enzml.mulan.MulanDataSetGenerator
```

**Direct Known Subclasses:**: AttributesFilteredDataSetGenerator

---

``` public class MulanDataSetGenerator extends DataSetGenerator ```

Class

**Version:**
:   8 Sep 2010

**Author:**
:   Luna De Ferrari luna.deferrari-at-ed.ac.uk

---

**Constructor Summary**

`MulanDataSetGenerator(MulanDataSetManager mulanDataSetManager,
uk.ac.ed.inf.utils.maputils.OneToManyMap<java.lang.String,java.lang.String> instanceAttributeMap,
uk.ac.ed.inf.utils.maputils.OneToManyMap<java.lang.String,java.lang.String> instanceClassMap)`


| **Method Summary** | |
| --- | --- |
| `int` | `getNumberOfAttributes()`             Get the number of distinct attribute values (all attribute values - class values) |
| `int` | `getNumberOfInstanceAttributeCouples()`             Get the number of distinct instance-attribute couples of values by subtracting the number of instance-class\_attribute values from the total number. |

**Methods inherited from class uk.ac.ed.inf.enzml.weka.DataSetGenerator**

`fillAndWriteInstancesConditional, fillInstances, getAttributes, getAttributesFactory, getClassName, getInstanceAttributeMap, getInstanceClassMap, getInstances, getInstancesFactory, getInstancesNames, getManager, getNumberOfClassValues, getNumberOfInstanceClassCouples, getNumberOfInstances, setInstancesNames`

**Methods inherited from class java.lang.Object**

`equals, getClass, hashCode, notify, notifyAll, toString, wait, wait, wait`

**Constructor Detail**

### MulanDataSetGenerator

```
public MulanDataSetGenerator(MulanDataSetManager mulanDataSetManager,
                             uk.ac.ed.inf.utils.maputils.OneToManyMap<java.lang.String,java.lang.String> instanceAttributeMap,
                             uk.ac.ed.inf.utils.maputils.OneToManyMap<java.lang.String,java.lang.String> instanceClassMap)
```


**Method Detail**

### getNumberOfAttributes

```
public int getNumberOfAttributes()
```

:   Get the number of distinct attribute values (all attribute values - class
    values)

    :   **Overrides:**: `getNumberOfAttributes` in class `DataSetGenerator`
    :   **Returns:**

---


### getNumberOfInstanceAttributeCouples

```
public int getNumberOfInstanceAttributeCouples()
```

:   Get the number of distinct instance-attribute couples of values by
    subtracting the number of instance-class\_attribute values from the total
    number.

    :   **Overrides:**: `getNumberOfInstanceAttributeCouples` in class `DataSetGenerator`


---


|  |  |  |
| --- | --- | --- |
| **Overview** \| **Package** \| **Class** \| **Use** \| **Tree** \| **Deprecated** \| **Index** \| **Help** | |  |
| **PREV CLASS**   **NEXT CLASS** | **FRAMES**    **NO FRAMES**     **All Classes** |  |
| SUMMARY: NESTED \| FIELD \| CONSTR \| METHOD | DETAIL: FIELD \| CONSTR \| METHOD |  |


---
